# Consuming foods with added oligofructose improves stool frequency: a randomised trial in healthy young adults

**DOI:** 10.1017/jns.2014.6

**Published:** 2014-04-30

**Authors:** Wendy J. Dahl, Arnelle R. Wright, Gretchen J. Specht, Mary Christman, Anne Mathews, Diederick Meyer, Thomas Boileau, Holly J. Willis, Bobbi Langkamp-Henken

**Affiliations:** 1 Food Science and Human Nutrition Department, University of Florida, 359 FSHN Building, Newell Drive, Gainesville, FL 32611, USA; 2 MCC Statistical Consulting LLC, 2219 NW 23rd Terrace, Gainesville, FL 32605, USA; 3 Sensus B.V., PO Box 1308, 4700 BH Roosendaal, The Netherlands; 4 General Mills Bell Institute of Health and Nutrition, 9000 Plymouth Avenue North, Golden Valley, MN 55427, USA

**Keywords:** Fibre, Oligofructose, Stool frequency, Gastrointestinal symptoms

## Abstract

The impact of oligofructose (OF) intake on stool frequency has not been clearly substantiated, while significant gastrointestinal (GI) symptoms have been reported in some individuals. The aim of the present study was to determine the effects of OF on stool frequency and GI symptoms in healthy adults. In an 8-week, randomised, double-blind, parallel-arm study, ninety-eight participants were provided with 16 g OF in yogurt and snack bars (twenty male and thirty female) or matching control foods (seventeen male and thirty-one female), to incorporate, by replacement, into their usual diets. Participants completed a daily online questionnaire recording stool frequency and rating four symptoms: bloating, flatulence, abdominal cramping and noise, each on a Likert scale from ‘0’ for none (no symptoms) to ‘6’ for very severe, with a maximum symptom intensity score of 24 (sum of severities from all four symptoms). Online 24 h dietary recalls were completed during pre-baseline and weeks 4, 6 and 8 to determine fibre intake. When provided with OF foods, fibre intake increased to 24·3 (sem 0·5) g/d from pre-baseline (12·1 (sem 0·5) g/d; *P* < 0·001). Stool frequency increased with OF from 1·3 (sem 0·2) to 1·8 (sem 0·2) stools per d in males and 1·0 (sem 0·1) to 1·4 (sem 0·1) stools per d in females during intervention weeks compared with pre-baseline (*P* < 0·05),but did not change for control participants (males: 1·6 (sem 0·2) to 1·8 (sem 0·2); females: 1·3 (sem 0·1) to 1·4 (sem 0·1)). Flatulence was the most commonly reported symptom. Mean GI symptom intensity score was higher for the OF group (3·2 (sem 0·3)) *v.* control (1·7 (sem 0·1)) (*P* < 0·01), with few participants reporting above moderate symptoms. No change in symptom intensity occurred over time. Consuming yogurt and snack bars with 16 g OF improves regularity in young healthy adults. However, GI symptoms, resulting from an increase in oligofructose intake, may not diminish with time.

In the USA, food processing and dietary preferences have resulted in fibre intakes far below recommendations. Americans currently consume less than half of the adequate intake for fibre^(^^1^^)^ (14 g fibre/4200 kJ (1000 kcal))^(^^2^^)^, an intake level that is associated with functional gastrointestinal disturbances such as constipation^(^^3^^)^. In an attempt to meet consumer demand for foods higher in fibre and to increase total fibre intake, foods with added fibre have become ubiquitous in the North American food supply, with fructans (inulin, oligofructose and fructo-oligosaccharides) being commonly added fibres^(^^4^^)^.

As many as 10 % of women and 4 % of men self-report constipation^(^^5^^)^; thus consumers may be seeking foods with added fibre to improve bowel habit. Although fructans have lower faecal bulking capacity compared with less fermentable fibres such as wheat bran^(^^6^^)^, there is evidence that inulin may improve bowel function in constipated adults^(^^7^^,^^8^^)^. However, it is not known if oligofructose, with its lower chain length, has a similar effect as longer-chain inulin, and if this effect can be demonstrated independent of low stool frequency.

Fermentation of undigested carbohydrate generates gas^(^^9^^)^, and oligosaccharides, due to their rapid fermentation^(^^10^^)^, may contribute to flatulence and bloating. Briet *et al.*^(^^11^^)^ evaluated gastrointestinal symptoms of fructo-oligosaccharide consumption and found that excessive flatulence was apparent at doses of >30 g/d, bloating at >40 g/d, and abdominal cramps and diarrhoea at >50 g/d. In a review of the safety and tolerance of fructans, it was suggested that up to 20 g per d of inulin and oligofructose are well tolerated^(^^12^^)^. However, more recent studies have suggested that the tolerance threshold may be as low as 10 g/d for inulin and 5 g/d for oligofructose^(^^13^^)^.

Comparing tolerance among studies is difficult, given that the gastrointestinal symptoms assessed and methodology used to rate severity differ. Carabin & Flamm^(^^12^^)^ noted that flatulence, bloating, abdominal distention and rumbling are commonly reported for inulin and oligofructose. Grabitske & Slavin^(^^14^^)^ reported that flatulence, distension, loose stools and stool frequency were observed in studies investigating tolerance to fructans. Bruhwyler *et al.*^(^^15^^)^ evaluated the tolerance of fructans, assessing flatulence, rumbling, bloating, abdominal pain, abdominal cramps and nausea, using visual analogue scales from 0 mm corresponding to ‘no symptoms’ to 100 mm corresponding to ‘unbearable symptoms’, as well as an assessment of stool frequency and stool consistency. More recently, Bonnema *et al.*^(^^13^^)^ assessed seven symptoms including gas/bloating, nausea, flatulence, gastrointestinal cramping, diarrhoea, constipation and gastrointestinal rumbling using a four-point scale (0 = none, 1 = mild, 2 = moderate, 3 = severe)^(^^13^^)^. The symptoms of flatulence, bloating/distension, noise/rumbling and gastrointestinal cramping are the most commonly reported symptoms to assess gastrointestinal tolerance of fructans, whereas nausea, not reported following oligofructose intake, may not be relevant for tolerance assessment. While both constipation and diarrhoea are commonly defined, in part, by stool frequency^(^^16^^)^, this objective measurement may be a more reliable indicator than self-reporting of these symptoms.

Foods with added fibre may provide a practical means of achieving fibre recommendations in North America. With widespread fructan intake, further evidence is needed related to the outcome measures of stool frequency and tolerance in healthy individuals. The aim of the present study was to determine the effect of consuming two foods per d, containing a total of 16 g oligofructose, on stool frequency and gastrointestinal symptoms in healthy adults compared with similar foods with no added fibre.

## Experimental methods

### Participants

Study participants were recruited from a university community through word of mouth, flyers, posters and announcements during autumn 2011. Inclusion criteria to examine both outcomes included: between the ages of 18 and 50 years, BMI between 23 and 30 kg/m^2^, a usual fibre intake of less than 20 g/d, weight stable (no loss or gain greater than 5 lb (2·3 kg) within the past 3 months) and habitual breakfast consumers (defined as eating breakfast within 2 h of waking at least 5 d per week). Exclusion criteria included: higher eating restraint (i.e. a score equal to or greater than 14 on the Eating Inventory questionnaire)^(^^17^^)^; postmenopausal (self-reported no menstrual period for 1 year); current smokers or tobacco users; not willing to discontinue prebiotic/fibre supplements or probiotic supplements; use of an antibiotic within 2 months before the study start; have known food allergies; have a physician-diagnosed gastrointestinal disease/condition other than gastro-oesophageal reflux disease, constipation or diverticular disease; were taking prescriptions other than oral contraceptives, seasonal allergy medications, cholesterol- or blood pressure-lowering medications, vitamins or minerals, and baby aspirin; having greater than two alcoholic beverages per d; participating in purposeful exercise for more than 5 h per week; or being a lactating or pregnant female. The present study was conducted according to the guidelines laid down in the Declaration of Helsinki and all procedures involving human subjects were approved by the University of Florida's Institutional Review Board 1 (IRB-01). Written informed consent was obtained from all participants.

### Experimental design

The study was an 8-week, randomised, double-blind, controlled, parallel-arm study. The present study was part of a larger study (*n* 200) designed to detect a 500 kJ difference between intervention groups with 80 % power, an α of 0·05, good blocking (0·4 correlations) and 15 % attrition rate. During the pre-baseline week, and during weeks 4, 6 and 8, participants recorded their daily dietary intake using the Automated Self-Administered 24 hour Recall (ASA24) system^(^^18^^)^. Baseline data were used to determine their average daily fibre and energy intake for the purposes of inclusion/exclusion and for stratification, respectively. Demographic data were also collected. Participants were randomised in blocks, with blocks being three different energy ranges for males (6·3–8·4 MJ (1500–2000 kcal), 8·4–10·9 MJ (2001–2600 kcal), >10·9 MJ (>2600 kcal)) and females (5·0–7·5 MJ (1200–1800 kcal), 7·5–10·0 MJ (1801–2400 kcal), >10·0 MJ (>2400 kcal)). The randomisation blocks and sealed envelopes containing subject assignments were prepared by the study statistician who did not have contact with the participants. Participants were provided with snack bars and yogurts to consume daily that, for the intervention group only, contained 16·4 g added oligofructose (8·4 g for study snack bars and 8·0 g for study yogurts). Table 1 gives the nutrient contents of the snack bars and yogurt. To provide a period of adaptation, participants were instructed to consume one snack bar per d during week 1, and one snack bar plus one yogurt from week 2 to the final week. Every 2 weeks, participants picked up the study foods for the upcoming weeks.

**Table 1.** Energy and nutrient content of yogurt and snack bars

Description	Weight (g)	Energy (kJ)	Carbohydrate (g)	Protein (g)	Fat (g)	Fibre (g)
Snack bar						
Control	38	599	31·8	1·5	1·0	0·5
Oligofructose	40	620	32·6	1·5	1·4	8·8*
Yogurt						
Control	170	448	19·5	6·8	0·2	0·1
Oligofructose	170	461	24·2	6·7	0·2	8·0

* Contained 8·4 g oligofructose.

Daily questionnaires, during the pre-baseline period and throughout the intervention period, were sent to each participant's email address or smart phone using Qualtrics Survey Software (Qualtrics Labs Inc.) along with electronic reminders to complete the questionnaires from study coordinators. The gastrointestinal symptom questionnaire was modified from Bonnema *et al.*^(^^13^^)^ by increasing the symptom rating scale to a seven-point scale recommended for subjective measures of symptoms^(^^19^^)^. Participants rated the intensity of gastrointestinal symptoms using a scale from 0 (none) to 6 (very severe) for bloating, flatulence, abdominal cramping and noises. The daily gastrointestinal symptom intensity score was defined as the sum of individual intensity scores from the four symptoms, with a maximum symptom severity score of 24. In addition, participants were asked to report daily stool frequency, the amount of each study food that they consumed, and whether they took an antibiotic. Once a participant reported that they started a course of antibiotics, their daily data from that point forward were not included in the analyses.

### Statistical analyses

Study data were analysed as intent to treat. Food fibre (from non-study foods) and total fibre were analysed using a linear mixed model with fixed effects of time period and treatment and a random effect to capture correlation among observations on the same subject within each time period, and a compound symmetry covariance structure for the residuals was completed to capture the repeated observations on each subject across periods.

The daily gastrointestinal symptom intensity score was averaged across each week for each subject. A generalised linear mixed model was then used to look for the effect of the intervention (control, oligofructose), sex and week of study (pre-baseline and intervention weeks 1 to 8) on gastrointestinal symptom intensity. A repeated-measures effect was included in the model to account for the repeated sampling on the same individual. Weekly scores were log-transformed to approximate a normal distribution and stabilise variance. The Kenward–Roger method^(^^20^^)^ was used to account for the effect of small sample bias in the repeated measures covariance estimates on hypothesis testing. The Tukey–Kramer method was used to control family-wise error rate in pairwise comparisons of means. Pairwise comparisons of weeks within each intervention were also performed in order to test for changes through time within an intervention, again adjusting for experiment-wise error rates using the Tukey–Kramer method. Daily scores for each symptom individually and stool frequencies were also analysed by averaging each week for each subject and then performing a generalised linear model as described above. A check of the assumptions indicated that bloating, cramping and noises required a log-transformation in order to obtain approximate normality. Probability of reporting at least one symptom (i.e. an intensity score >0 for at least one symptom) and the probability of reporting all four symptoms were estimated by analysing daily indicator variables (for example, yes = 1 if any symptoms reported and = 0 otherwise) using logistic repeated-measures mixed models with fixed effects of study group, sex and week of study and a repeated-measures effect to allow for repeated observations on the same individual. Mean differences in demographic data, daily intake of study foods and stool number by study group and sex were analysed using a two-way ANOVA and the Holm–Sidak All Pairwise Multiple Comparison Procedure. Unless stated otherwise, data are presented as mean values with their standard errors, with significance denoted at *P* < 0·05. Statistical analyses were completed using SAS (version 9.2; SAS Institute Inc.) or SigmaPlot (version 11.0, 2008; Systat Software Inc.).

## Results

**Fig. 1.**
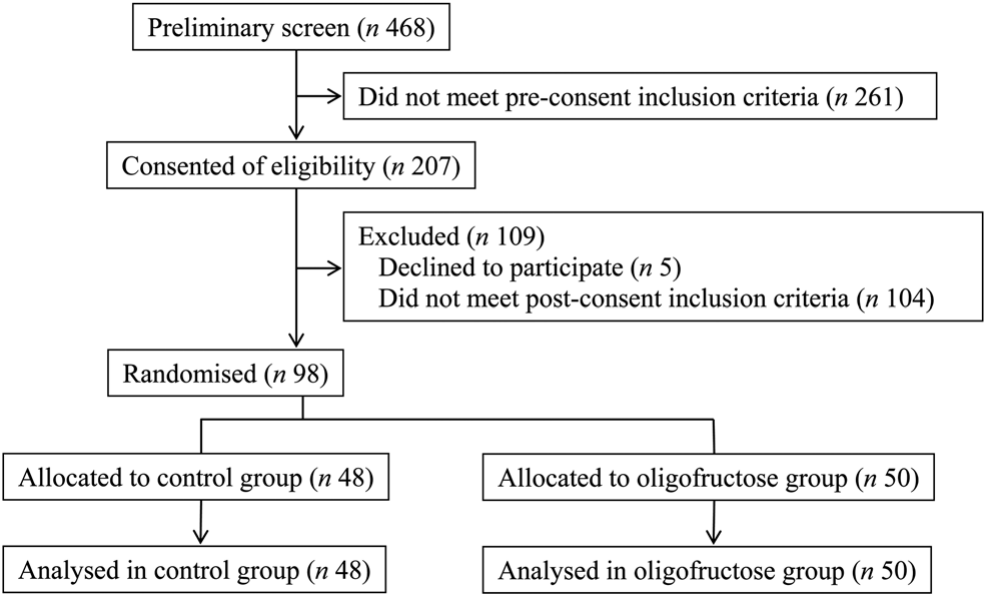
Participant flow diagram.

Informed consent was obtained from 207 potential participants (Fig. 1). Of these, 109 did not meet inclusion/exclusion criteria, for reasons such as excess exercise (*n* 10), a usual fibre intake of over 20 g/d (*n* 22), BMI out of range (*n* 25), disordered eating (*n* 20), under-reporting of energy intake (*n* 16), and other (*n* 11). Of the ninety-eight participants randomised, ninety-seven completed all 56 d of the study protocol. One participant voluntarily withdrew from the study after 6 d due to excessive gastrointestinal disturbances. This participant consumed the oligofructose-containing bar for the first 3 d only and reported an average gastrointestinal symptom intensity score of 11 of a possible 24. During the study, seven participants began a course of antibiotics and their data were not included in the analyses from the first day of antibiotics until the end of the study. Of these participants, five were receiving the control foods, and antibiotics were started on day 16, 22, 34, 39 and 40. Two of the participants were consuming the oligofructose-containing foods, and antibiotics were started on days 22 and 41. Due to sex differences in gastrointestinal symptom data and stool frequency, data are reported by study group and sex.

The majority of the participants were white. Mean age and BMI were not different between study groups or sex (Table 2). Overall, participants completed 95 (sem 1) % of the daily questionnaires and reported that they consumed 96 (sem 1) % of the study foods for a mean intake of 15·4 (sem 0·3) g oligofructose/d for those randomised to the oligofructose-containing foods. Mean daily intake of oligofructose from the study foods was not different between sexes (Table 2).

**Table 2.** Participant characteristics, study food and oligofructose intake* (Mean values with their standard errors, or numbers of subjects and percentages)

	Males				Females			
	Control		Oligofructose		Control		Oligofructose	
	Mean	sem	Mean	sem	Mean	sem	Mean	sem
Subjects (*n*)	17		20		31		30	
Age (years)	24·2	1·6	25·3	1·5	24·6	1·2	22·9	1·2
Daily questionnaires completed (%)	98·6	2·2	89·1	2·0	96·4	1·6	97·1	1·7
Race/ethnicity								
White								
*n*	11		13		15		13	
%	65		65		48		43	
Hispanic								
*n*	4		3		3		5	
%	24		15		10		17	
Asian								
*n*	2		2		4		3	
%	12		10		13		10	
Black								
*n*	0		2		6		9	
%	0		10		19		30	
Other								
*n*	0		0		3		0	
%	0		0		10		0	
BMI (kg/m^2^)	25·3	0·5	25·9	0·5	25·4	0·4	25·5	0·4
Study foods per d (%)	97·3	2·2	95·3	2·0	97·1	1·6	94·0	1·6
Study bars per d (%)	96·9	2·5	94·5	2·3	99·4	1·9	96·5	1·9
Study yogurts per d (%)	97·8	3·8	96·1	3·5	94·4	2·8	92·6	2·9
Oligofructose intake (g/d)†	0		15·6	0·4	0		15·3	0·3

* *P* values for sex, intervention group, or the interaction between sex and intervention group effects were examined with no significant sex × intervention interactions observed.

† Added oligofructose in bars and yogurts is from study weeks 2 to 8.

**Fig. 2.**
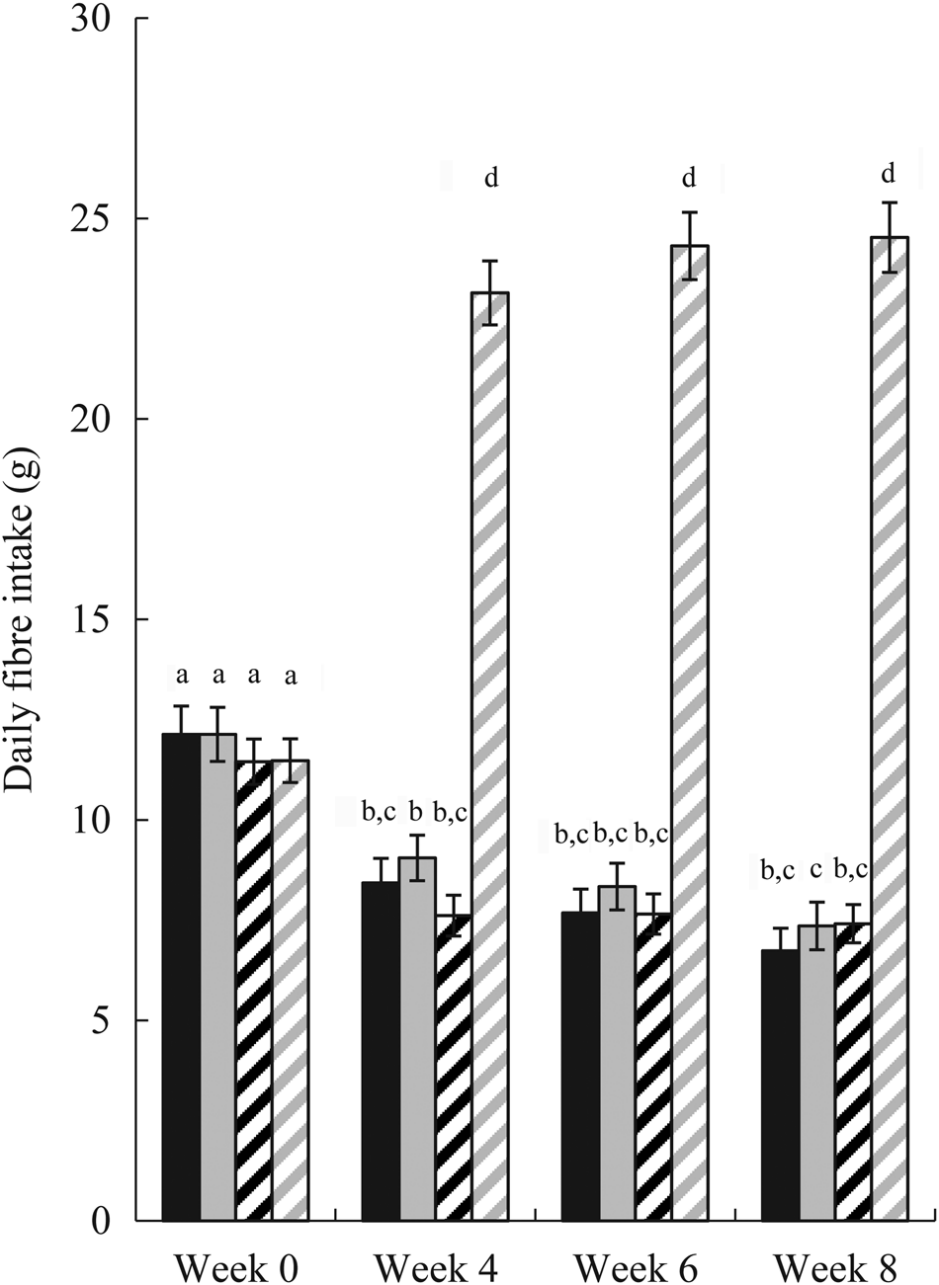
Daily fibre intake from all foods (total fibre) or non-study foods (food fibre) by study week in individuals receiving study foods without oligofructose (control) or with oligofructose (intervention). ■, Food fibre (control); 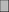, total fibre (control); 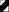, food fibre (intervention); 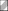, total fibre (intervention). Values are means, with standard errors represented by vertical bars. ^a,b,c,d^ Mean values with unlike letters were significantly different (*P* < 0·01).

Mean total fibre intakes were not significantly different between the control and oligofructose groups during pre-baseline (week 0) (Fig. 2). However, when comparing mean total fibre intake between the two groups during the intervention period (weeks 4, 6 and 8), the oligofructose group consumed considerably more fibre over time than the control group, as expected (*P* < 0·001). While the oligofructose group increased their total fibre intake over the duration of the study, the total fibre intake of the control group was consistently lower from baseline to study completion.

**Fig. 3.**
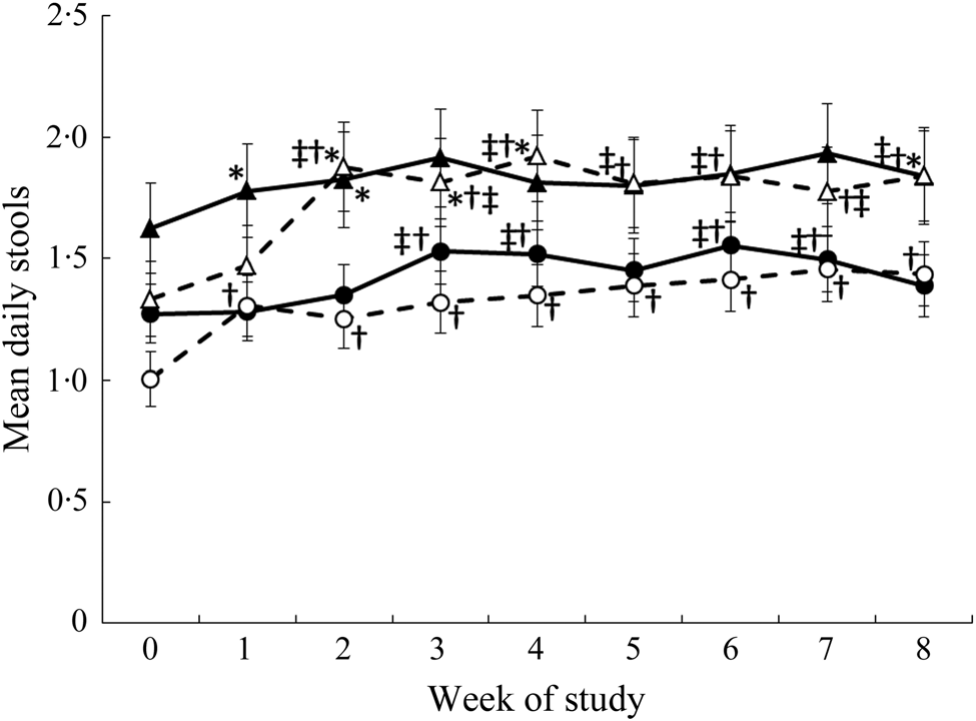
Mean daily stool frequency by week. ●, Females (control); ○, females (oligofructose); ▴, males (control); ▵, males (oligofructose). Values are means, with standard errors represented by vertical bars. Main effects: intervention group (I), *P* = 0·5026; sex (S), *P* = 0·0067; week (W), *P* < 0·0001; I × W, *P* = 0·0044; I × S × W, *P* = 0·0170. * Mean value was significantly different from that of females in the same group (*P* < 0·05). † Mean value was significantly different from that at pre-baseline (week 0) for the same group (*P* < 0·05). ‡ Mean value was significantly different from that at week 1 for the same group (*P* < 0·05).

There was a significant three-way interaction between the intervention, sex and week (*P* = 0·017) for mean stool frequency (Fig. 3). An increase in stool frequency was observed in females in the oligofructose group after the addition of the first study food, i.e. from week 1 of the intervention to week 8 compared with the pre-baseline week 0 (Fig. 3). Daily stool frequency increased for males consuming the oligofructose study foods at week 2 (i.e. after the addition of the second study food) to week 8 of the intervention compared with the pre-baseline week (Fig. 3).

**Fig. 4.**
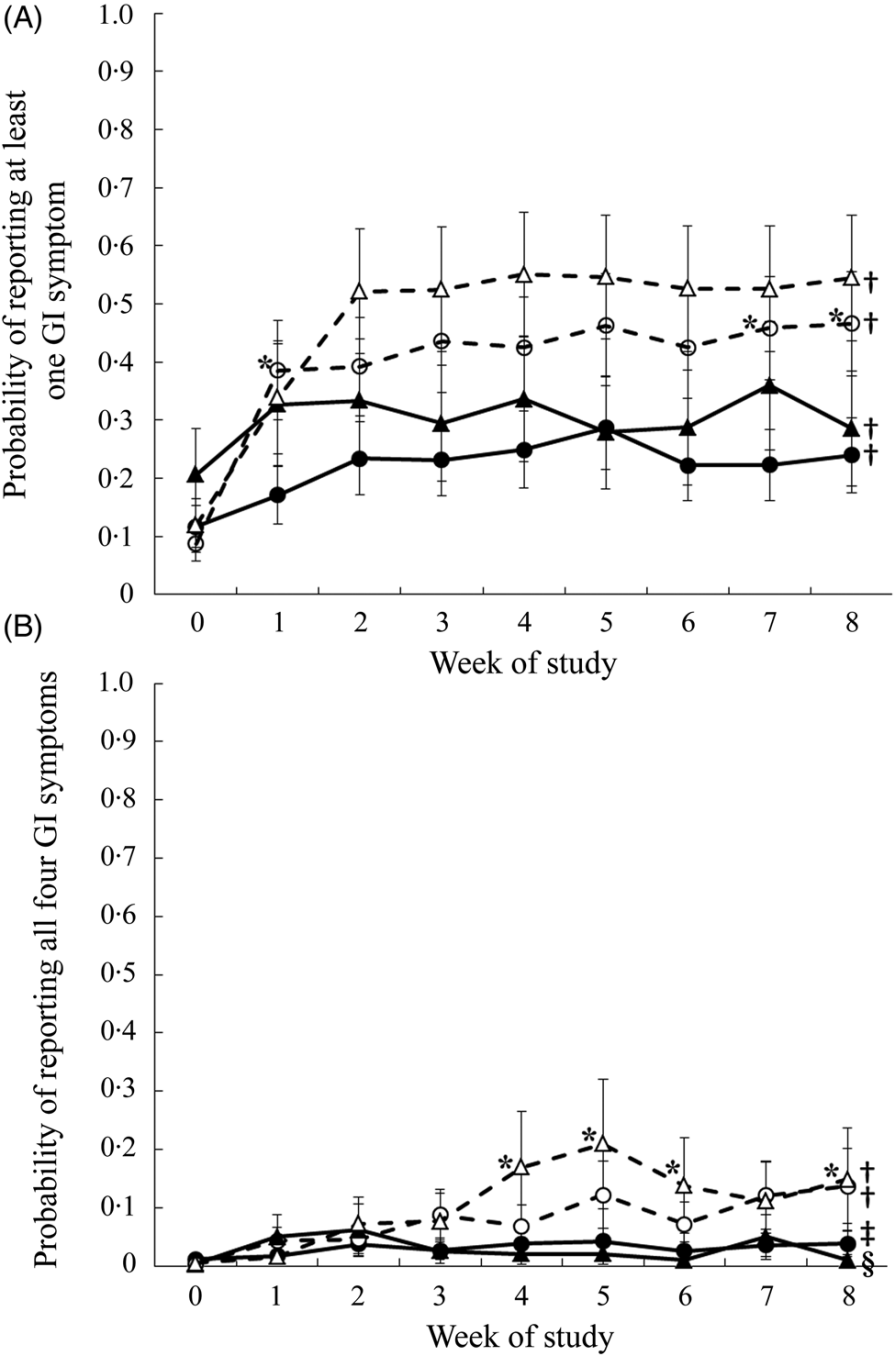
Probability of reporting at least one of four gastrointestinal (GI) symptoms (flatulence, bloating, abdominal cramping, or noises) (A) or all four symptoms (B) in 1 d during the pre-baseline (week 0) period and 8 weeks of intervention. Daily data were averaged across each week for each participant. ●, Females (control); ○, females (oligofructose); ▴, males (control); ▵, males (oligofructose). Values are least squares means, with standard errors represented by vertical bars. For (A), main effects: intervention group (I), *P* = 0·0718; sex (S), *P* = 0·3409; week (W), *P* < 0·0001; I × S × W, *P* < 0·0001. For B, main effects: intervention group (I), *P* = 0·0846; sex (S), *P* = 0·9355; week (W), *P* < 0·0001; I × S × W, *P* = 0·0032. * Mean value was significantly different from that of the control for the same sex (*P* < 0·05). † Mean values for all the weeks were significantly different from that for the pre-baseline week (*P* < 0·05). ‡ Mean values for the study weeks 2, 3, 4, 5, 7 and 8 were significantly different from that for the pre-baseline week (*P* < 0·05). § Mean values for the study weeks 1, 2, 3 and 7 were significantly different from that for the pre-baseline week (*P* < 0·05).

The probability of reporting at least one symptom or all four symptoms was considered. There was a significant (*P* < 0·0001) three-way interaction among intervention group, sex and week on the probability of reporting at least one out of four symptoms. Males consuming both the oligofructose and the control foods had a higher probability of reporting at least one symptom during all study weeks compared with that reported during the pre-baseline period. There was no difference in the probability of reporting at least one symptom at any time point for male participants consuming the oligofructose *v.* control foods (Fig. 4(A)). Similarly, females in both study groups had a higher probability of reporting at least one symptom during the study weeks compared with the pre-baseline period. However, during study weeks 1, 7 and 8, females consuming the oligofructose had a higher probability of reporting at least one symptom compared with females consuming the control foods.

There was also a significant (*P* = 0·003) three-way interaction among intervention group, sex and week on the probability of reporting all four symptoms (Fig. 4(B)). Compared with the pre-baseline period, males had a higher probability of reporting all four symptoms during weeks 1 to 8 when consuming the oligofructose and weeks 1, 2, 3 and 7 when consuming the control foods. During study weeks 4, 5, 6 and 8, males consuming the oligofructose had a higher probability of reporting all four symptoms than males consuming the control foods. Throughout the pre-baseline period and the 8-week intervention, there was less than a 15 % probability that females reported all four symptoms, and there was no difference between study groups. Compared with the pre-baseline period, females consuming oligofructose had a significantly higher probability of reporting all four symptoms during weeks 1 to 8 of the intervention, and those in the control group had a higher probability during all weeks except weeks 1 and 6.

**Fig. 5.**
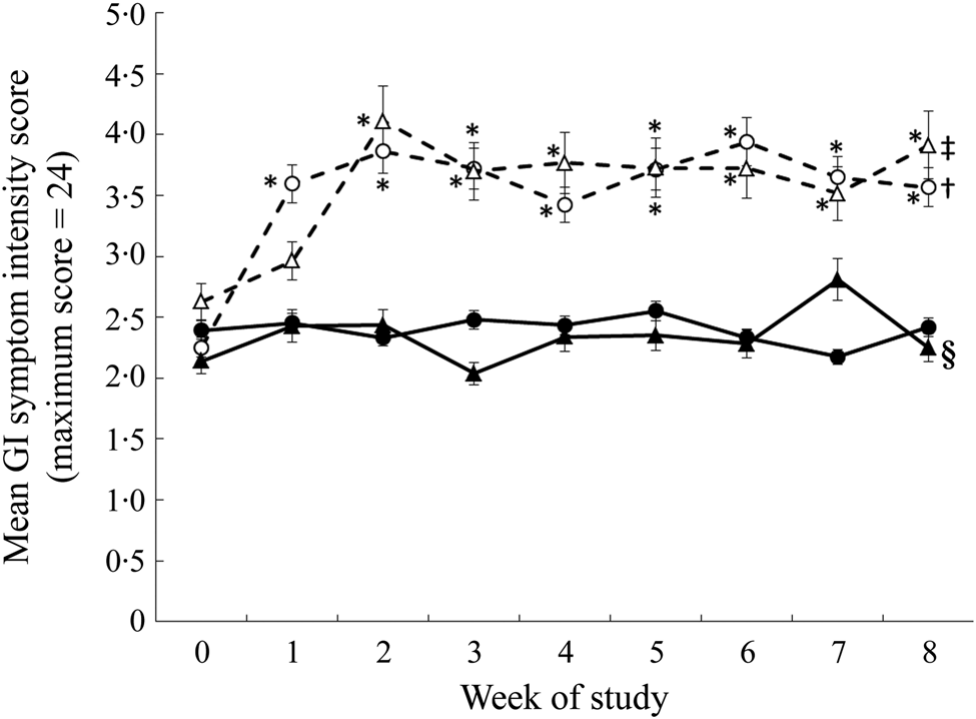
Gastrointestinal (GI) symptom intensity scores during the pre-baseline (week 0) and 8-week intervention periods in males and females consuming the oligofructose-containing or control foods. The daily GI symptom intensity score represents the sum of symptom intensities (0 = no symptom to 6 = very severe symptoms) for flatulence, bloating, abdominal cramping and stomach noises averaged across each week for each participant. ●, Females (control); ○, females (oligofructose); ▴, males (control); ▵, males (oligofructose). Data were analysed as log-normally distributed. Values are back-transformed least squares means, with standard errors represented by vertical bars. Main effects: intervention group (I), *P* = 0·0009; sex (S), *P* = 0·9272; week (W), *P* < 0·0001; I × S × W, *P* = 0·0095. * Mean value was significantly different from that of the control for the same sex (*P* < 0·05). † Mean values for all the weeks were significantly different from that for the pre-baseline week (*P* < 0·05). ‡ Mean values for the study weeks 2 to 8 were significantly different from that for the pre-baseline week (*P* < 0·05). § Mean value for study week 7 was significantly different from that for the pre-baseline week (*P* < 0·05).

There was no difference in the gastrointestinal symptom intensity score during the pre-baseline (week 0) period between sex or intervention assignment. There was a significant effect of oligofructose on the mean symptom intensity (Fig. 5), with those consuming oligofructose reporting a higher mean symptom intensity score (i.e. sum of intensity from all four symptoms) during the intervention period. Mean daily symptom intensity score was significantly higher for the oligofructose group during all 8 weeks of the intervention compared with the pre-baseline period. There were no differences in symptom intensity score between intervention weeks with the exception of weeks 1 and 2 where the symptom intensity score was significantly higher at week 2 *v.* week 1 for those consuming the oligofructose. There was a three-way interaction of study group, week and sex (*P* = 0·01; Fig. 5). For males consuming the oligofructose, the symptom intensity score did not increase compared with the pre-baseline period until week 2 with the addition of the second oligofructose-containing study food (*P* < 0·0001) and remained elevated to week 8 (Fig. 5). The symptom intensity score for males consuming the control foods was not different from the pre-baseline period with the exception of study week 7 when a higher score was reported. The symptom intensity score was significantly higher for males consuming the oligofructose *v.* control foods during study weeks 2 to 6 and week 8. For females consuming the oligofructose, the symptom intensity score significantly increased (*P* < 0·0001) at study week 1 when they were consuming only one of the study foods per d and was constant throughout the remainder of the 8-week study period even after the addition of the second study food. For females consuming the control foods the symptom intensity score was similar to that reported during the pre-baseline period. The symptom intensity score was significantly (*P* < 0·05) higher for women consuming the oligofructose *v.* control foods from study week 1 to 8.

Mean daily symptom intensity scores for flatulence and noises were significantly higher with oligofructose (Table 3). Males and females consuming oligofructose reported higher symptom intensity scores for flatulence during week 8 of the intervention compared with those consuming the control foods. The symptom intensity scores for noises were significantly higher at weeks 2, 4 and 5 of the intervention in participants consuming the oligofructose *v.* the control foods. Of 5653 total participant days reported, moderate (defined as > 3 intensity rating) bloating was reported in 1·4 % of control participant days compared with 3·0 % of oligofructose participant days. Moderate flatulence was reported in 2·3 % of control participant days *v.* 12·0 % treatment participant days. Moderate cramping was reported in 1·0 % control participant days *v.* 2·5 % of treatment participant days, and moderate noises were reported in 0·61 % of control participant days *v.* 3·1 % treatment participant days.

**Table 3.** Gastrointestinal symptom intensities in males and females consuming the control or oligofructose-containing foods (Least squares means (LSmeans) with their standard errors)

	Males				Females				
	Control		Oligofructose		Control		Oligofructose		
	LSmean	sem	LSmean	sem	LSmean	sem	LSmean	sem	P*
Subjects (*n*)	17		20		31		30		
Gastrointestinal symptom intensities per d†									
Flatulence									I: <0·0001
Pre-baseline	0·7	0·1	0·7^a^	0·2	0·7^b^	0·1	0·6^c^	0·2	S: 0·372
Intervention (week 8)	1·0^d^	0·2	2·1^a,d^	0·3	0·8^b,e^	0·2	1·6^c,e^	0·2	W: <0·0001
									I × S × W: 0·086
Bloating									I: 0·038
Pre-baseline	0·3	0·1	0·2	0·1	0·2	0·1	0·2	0·1	S: 0·852
Intervention (week 8)	0·4	0·2	0·8	0·3	0·4	0·1	0·9	0·2	W: 0·226
									I × S × W: 0·469
Abdominal cramping									I: 0·166
Pre-baseline	0·1^a^	0·1	0·2	0·1	0·3^a^	0·1	0·2	0·1	S: 0·695
Intervention (week 8)	0·2^b^	0·1	0·8	0·3	0·3^b^	0·1	0·7	0·2	W: 0·759
									I × S × W: 0·087
Noises									I: 0·076
Pre-baseline	0·3	0·1	0·3	0·1	0·4^a^	0·1	0·3	0·1	S: 0·808
Intervention (week 8)	0·3	0·2	0·8	0·3	0·4^a^	0·1	0·6	0·2	W: 0·611
									I × S × W: 0·003

^a,b,c,d,e^ Mean values within rows (sex and treatment) and columns (week) with similar letters were significantly different (*P* < 0·05). Values without superscripts were not different from any other values within rows or columns.

* *P* values for intervention group (I), sex (S), week (W) and their interaction.

† The mean daily gastrointestinal symptom intensity score represents the sum of symptom intensities (0 = no symptom to 6 = very severe symptoms).

On the final questionnaire participants were asked to report which study group they thought they were in (i.e. ‘fibre-supplemented group’ or ‘non-fibre-supplemented group’). Of those participants consuming the control foods, 51 % thought they were in the control group and 49 % thought they were consuming the fibre-containing foods. Of those participants randomised to the oligofructose-supplemented foods a significantly larger proportion (78 %; *P* = 0·005) thought they were in the fibre-supplemented group. When the responses from males and females were examined separately, males were not able to correctly guess their assigned group (*P* = 0·16). A significantly larger proportion of females (76 %) were able to correctly guess that they were consuming the fibre-supplemented foods (*P* = 0·03).

## Discussion

With the goal of recruiting participants with typical North American fibre intakes, we excluded individuals with habitual fibre intakes of greater than 20 g/d. The mean fibre intake during pre-baseline was 12·1 (sem 0·5) g/d, somewhat below the estimated US fibre intake^(^^1^^)^. The addition of two snacks with added oligofructose increased the mean total fibre intake to 24·3 (sem 0·5) g/d, which nearly achieved the adequate intake for fibre in this group. Providing low-fibre foods to the control group may have displaced higher-fibre foods from their usual diet, as their fibre intakes decreased during the intervention. The high compliance reported in the present study, similar in both control and oligofructose groups, provides evidence that the oligofructose-fortified foods were acceptable^(^^21^^)^.

Incorporating foods with 16 g oligofructose/d into the diets of healthy young adults resulted in increased stool frequency. This is in contrast with the findings of Slavin & Feirtag^(^^22^^)^, who reported no change in stool frequency or other indicators of bowel function, i.e. stool weight, intestinal transit time and consistency, with 20 g inulin in young healthy males (*n* 12) consuming a controlled diet, suggesting that their study may have been underpowered. However, the findings of the present study are in agreement with two studies demonstrating improved bowel function with chicory inulin with constipated subjects^(^^7^^,^^8^^)^.

The present study confirms that a significant increase in gastrointestinal symptoms, particularly flatulence, results with the consumption of oligofructose, a finding that is in agreement with previous studies^(^^13^^)^. One participant withdrew from the study due to symptoms confirming that perhaps about 1 % of healthy individuals may be ‘highly sensitive’ to rapidly fermentable fibres^(^^23^^,^^24^^)^. For the remaining participants, ‘above moderate’ symptoms were reported on few days. In the oligofructose group, flatulence, the most commonly reported symptom, was reported at >3 on only 12 % of days. In reference to limitations, the present study included only healthy young participants. Older adults and children may respond differently to a similar intake of oligofructose.

Although it is commonly described that individuals adapt to increased fibre intake over time with decreasing symptoms^(^^24^^)^, the results of the present study challenge this assumption. Reported symptoms with oligofructose consumption maintained the same level of intensity over time, and no adaptation occurred. This finding is in agreement with that of Stone-Dorshow & Levitt^(^^25^^)^ who found that gas symptoms with an intake of 10 g fructo-oligosaccharides did not change over a 12 d period^(^^25^^)^. Adaptation, if defined as a decrease in gastrointestinal symptoms such as flatulence over time, would be dependent on the microbiota shifts that occur with the feeding of a fibre and resulting substrate availability and fermentation. However, adaptation of the colonic microbiota is nebulous given that it is a dynamic system and defining an endpoint of adaptation is uncertain. Significant shifts in bacterial populations, that may make an impact on gas production and, therefore, gastrointestinal symptoms, generally occur within the first 2 weeks of intervention^(^^26^^)^. The present study, given its 8-week length, clearly demonstrates no gastrointestinal symptom adaptation to oligofructose.

## 

### Conclusions

Oligofructose, with its positive impact on bowel habit when provided at 16 g/d, has potential for improving population fibre intakes. On most participant days, oligofructose produced mild to moderate symptoms. However, as one individual experienced enough discomfort to stop the study, labelling and identification of foods with added fibre is prudent.

## References

[1] United States Department of Agriculture, Agricultural Research Service (2006) What We Eat in America, National Health and Nutrition Examination Survey (NHANES) 2003–2006. http://www.ars.usda.gov/SP2UserFiles/Place/12355000/pdf/0506/usual_nutrient_intake_dietary_fiber_2003-06.pdf (accessed February 2013).

[2] Institute of Medicine, Food and Nutrition Board (2005) Dietary Reference Intakes for Energy, Carbohydrate, Fiber, Fat, Fatty Acids, Cholesterol, Protein, and Amino Acids, pp. 339–421 Washington, DC: The National Academies Press

[3] Jamshee N, Lee Z-E & Olden KW (2011) Diagnostic approach to chronic constipation in adults. Am Fam Physician 84, 299–306 21842777

[4] Fungwe TV, Bente L & Hiza H (2007) The Food Supply and Dietary Fiber: Its Availability and Effect on Health*: Nutrition Insight 36*. Alexandria, VA: USDA Center for Nutrition Policy and Promotion

[5] Markland AD, Palsson O, Goode PS, (2013) Association of low dietary intake of fiber and liquids with constipation: evidence from the National Health and Nutrition Examination Survey (NHANES). Am J Gastroenterol 108, 796–803 23567352 10.1038/ajg.2013.73 PMC3786707

[6] Nyman M (2002) Fermentation and bulking capacity of indigestible carbohydrates: the case of inulin and oligofructose. Br J Nutr 87, Suppl. 2, S163–S168 12088514 10.1079/BJNBJN/2002533

[7] Den Hond E, Geypens B & Ghoos Y (2000) Effect of high performance chicory inulin on constipation. Nutr Res 20, 731–736

[8] Marteau P, Jacobs H, Cazaubiel M, (2011) Effects of chicory inulin in constipated elderly people: a double-blind controlled trial. Int J Food Sci Nutr 62, 164–167 21091293 10.3109/09637486.2010.527323

[9] Cummings JH, Beatty ER, Kingman SM, (1996) Digestion and physiological properties of resistant starch in the human large bowel. Br J Nutr 75, 733–747 8695600 10.1079/bjn19960177

[10] Stewart M, Timm D & Slavin J (2008) Fructooligosaccharides exhibit more rapid fermentation than long-chain inulin in an *in vitro* fermentation system. Nutr Res 28, 329–334 19083428 10.1016/j.nutres.2008.02.014

[11] Briet F, Achour L, Flourie B, (1995) Symptomatic response to varying levels of fructo-oligosaccharides consumed occasionally or regularly. Eur J Clin Nutr 49, 501–507 7588500

[12] Carabin IG & Flamm WG (1999) Evaluation of safety of inulin and oligofructose as dietary fiber. Regul Toxicol Pharmacol 30, 268–282 10620476 10.1006/rtph.1999.1349

[13] Bonnema AL, Kolberg LW, Thomas W, (2010) Gastrointestinal tolerance of chicory inulin products. J Am Diet Assoc 110, 865–868 20497775 10.1016/j.jada.2010.03.025

[14] Grabitske HA & Slavin JL (2009) Gastrointestinal effects of low-digestible carbohydrates. Crit Rev Food Sci Nutr 49, 327–360 19234944 10.1080/10408390802067126

[15] Bruhwyler J, Carreer F, Demanet E, (2009) Digestive tolerance of inulin-type fructans: a double-blind, placebo-controlled, cross-over, dose-ranging, randomized study in healthy volunteers. Int J Food Sci Nutr 60, 165–175 18608562 10.1080/09637480701625697

[16] Wilkins T, Pepitone C, Alex B, (2012) Diagnosis and management of IBS in adults. Am Fam Physician 86, 419–426 22963061

[17] Garner DM (2004) Eating Disorder Inventory-3. Professional Manual. Lutz, FL: Psychological Assessment Resources, Inc

[18] United States National Institutes of Health (2012) ASA24 Automated Self-Administered 24-hour Recall. http://riskfactor.cancer.gov/tools/instruments/asa24/resources/portal.html (accessed February 2013).

[19] Jaeschke R, Singer J & Guyatt GH (1990) A comparison of seven-point and visual analogue scales. Data for a randomized trial. Control Clin Trials 11, 43–51 2157581 10.1016/0197-2456(90)90031-v

[20] Kenward MG & Roger JH (1997) Small sample inference for fixed effects from restricted maximum likelihood. Biometrics 53, 983–997 9333350

[21] Niness KR (1999) Inulin and oligofructose: what are they? J Nutr 129, 1402S–1406S 10395607 10.1093/jn/129.7.1402S

[22] Slavin J & Feirtag J (2011) Chicory inulin does not increase stool weight or speed up intestinal transit time in healthy male subjects. Food Funct 2, 72–77 21773588 10.1039/c0fo00101e

[23] Cummings JH, Macfarlane GT & Englyst HN (2001) Prebiotic digestion and fermentation. Am J Clin Nutr 73, 415S–420S 11157351 10.1093/ajcn/73.2.415s

[24] Kolida S, Meyer D & Gibson GR (2007) A double-blind placebo-controlled study to establish the bifidogenic dose of inulin in healthy humans. Eur J Clin Nutr 61, 1189–1195 17268410 10.1038/sj.ejcn.1602636

[25] Stone-Dorshow T & Levitt M (1987) Gaseous response to digestion of poorly absorbed fructooligosaccharides sweetener. Am J Clin Nutr 46, 61–65 3604970 10.1093/ajcn/46.1.61

[26] Mai V, Katki HA, Harmsen H, (2004) Effects of a controlled diet and black tea drinking on the fecal microflora composition and the fecal bile acid profile of human volunteers in a double-blinded randomized feeding study. J Nutr 134, 473–478 14747691 10.1093/jn/134.2.473

